# NADH-Mediated Gene Expression in *Streptococcus pneumoniae* and Role of Rex as a Transcriptional Repressor of the Rex-Regulon

**DOI:** 10.3389/fmicb.2018.01300

**Published:** 2018-06-19

**Authors:** Muhammad Afzal, Sulman Shafeeq, Oscar P. Kuipers

**Affiliations:** ^1^Molecular Genetics Group, Groningen Biomolecular Sciences and Biotechnology Institute, University of Groningen, Groningen, Netherlands; ^2^Bioinformatics and Biotechnology, Government College University, Faisalabad, Pakistan; ^3^Karolinska Institutet, Solna, Sweden

**Keywords:** NADH, Rex, PncB, pneumococcus, GaPN

## Abstract

Nicotinamide adenine dinucleotides (NAD(H)) play a vital role in various biological processes, including keeping the cellular redox balance. In this study, we investigate the regulatory responses of *Streptococcus pneumoniae* D39 to NADH and characterize the role of the Rex protein as a transcriptional repressor of the *gapN, fba, pncB, adhB2, gap*, and *adhE* genes. Transcriptomic analysis was used to observe the response of *S. pneumoniae* D39 to NADH. Our microarray studies revealed elevated expression of various genes/operons involved in transport and biosynthesis of niacin (*gapN, fba, pncB, adhB2, gap*, and *adhE*). Promoter *lacZ*-fusion assays and microarray studies with the *rex* mutant revealed the role of Rex as a transcriptional repressor of *gapN, fba, pncB, adhB2, gap*, and *adhE* involved in niacin uptake and biosynthesis, in the presence of NADH. We predict the operator site (5′-TTGTKAWAAWWTTCACAA-3′) of Rex in the regulatory regions of Rex-regulated genes that was subsequently validated by promoter mutational experiments.

## Introduction

*Streptococcus pneumoniae* is an important human nasopharyngeal pathogen and causes several infections leading to over a million deaths around the globe each year (Ispahani et al., 2004; O’Brien et al., 2009). Bacteria have to make use of the nutrients present in their surroundings, in addition to the virulence factors they possess, to successfully colonize and spread infections (Phillips et al., 1990; Titgemeyer and Hillen, 2002). Vitamins and co-factors are among the most important compounds needed for bacterial growth.

Nicotinamide Adenine Dinucleotide (NAD) participates in several cellular processes as an important cofactor for living organisms (Ishino et al., 1986; Chiarugi et al., 2012; Patel et al., 2014). NAD^+^ is an oxidized form of NAD, whereas NADH is its reduced form and niacin (nicotinic acid) acts as a precursor of NAD and NADP (Jurtshuk, 1996). The role of niacin in the regulatory mechanisms of NiaR and on global gene expression in *S. pneumoniae* has been revealed in our previous study (Afzal et al., 2017). Several genes, including *nadC, niaX, fba, pnuC, rex, pncB, gapN, gap, adhB2*, and *adhE*, were differentially expressed and role of NiaR as a transcriptional repressor of *nadC, pnuC*, and *niaX* was demonstrated in our study (Afzal et al., 2017).

There are several processes in bacteria to reduce NAD^+^ to NADH, for instance respiration, which involves either glycolysis and the tricarboxylic acid cycle or fermentation (Jurtshuk, 1996). The regeneration of NAD^+^ is an important process in bacteria for continued carbohydrate catabolism (Marquis, 1995; Higuchi et al., 1999). Aerobically, and when glucose is abundant, the intracellular concentration of fructose 1,6-bisphosphate increases, which induces the expression of lactate dehydrogenase (*ldh*) (Abbe et al., 1982). Elevated *ldh* expression levels keep the redox balance required for continuous glycolytic activity, as pyruvate gets converted into lactate and NADH is oxidized to NAD^+^ (Abbe et al., 1982). Bacteria also have the ability to use NAD^+^ in different forms of dehydrogenases when metabolizing aldehydes and alcohols (Temple et al., 1994; Nobelmann and Lengeler, 1996; Luong et al., 2015).

Rex was recognized in *Streptomyces coelicolor* as a redox-associated transcriptional regulator of *nuoA*-*nuoN, hemACD*, and *cydABCD* (Brekasis and Paget, 2003). Since then, the Rex protein has been identified in numerous other model microorganisms including *Staphylococcus aureus, Bacillus subtilis, Enterococcus faecalis*, and *Streptococcus mutans*. The Rex regulon has been experimentally characterized in the model hyperthermophilic bacterium *Thermotoga maritima* (Ravcheev et al., 2012). In *Desulfovibrio vulgaris* Hildenborough, a model Gram-negative bacterium for studying sulfate reduction, Rex has been described to be regulated by NADH and acts as a repressor of sulfate adenylyl transferase (Christensen et al., 2015). Moreover, Rex has been shown to be a global redox-sensing transcriptional regulator in *Clostridium kluyveri* and its role in the regulation of oxidative stress tolerance and alcohol production has been studied in *Clostridium acetobutylicum* (Zhang et al., 2014; Hu et al., 2016). Furthermore, Rex-associated transcriptional modulation has been attributed to species-specific genetic targets of various organisms and a putative Rex-binding motif and individual sites have been predicted previously (Ravcheev et al., 2012). Rex monitors the transcription of genes associated with fermentation, amino acid metabolism, and nitrate/nitrite respiration in *S. aureus* (Pagels et al., 2010). Rex regulates the transcription of genes related to oxidative stress response, biofilm formation, and fermentation in cariogenic *S. mutans* (Baker et al., 2014; Bitoun et al., 2011, 2012). Rex-deficiency in *S. mutans* leads to enhanced sensitivity to exogenous H_2_O_2_ and higher end-point pH values of stationary-phase culture medium (Baker et al., 2014; Bitoun et al., 2011, 2012). Moreover, absence of Rex results in diminished growth of aerated cultures, which can be restored by adding catalase to the medium. These observations suggest that a *rex*-deletion mutant has reduced capability to deal with oxidative stress (Mehmeti et al., 2011; Vesić and Kristich, 2013).

This study reveals the impact of NADH on the transcriptome of *S. pneumoniae* and characterizes the Rex regulon. We demonstrate that Rex represses the expression of several genes/operons involved in NADH uptake and consumption. Furthermore, we predict the putative operator site (5′- TTGTKAWAAWWTTCACAA -3′ such that W = A/T and K = G/T) of Rex in the promoter regions of its regulon genes that is also confirmed by promoter mutational experiments.

## Experimental Methods

### DNA Techniques, Growth Conditions, and Bacterial Strains

Bacterial strains and plasmids are tabulated in Supplementary Table S1 and primers in Supplementary Table S2. *S. pneumoniae* D39 chromosomal DNA was used to perform PCR amplification (Lanie et al., 2007). Growth of *S. pneumonia*e D39 and DNA manipulation techniques were performed as mentioned before (Kloosterman et al., 2006; Afzal et al., 2014). We used *S. pneumoniae* D39 derivatives grown in a chemically defined medium (CDM) supplemented either with 0.5 mg/ml NADH or without NADH for β-galactosidase assays. CDM was prepared without niacin. For pneumococcal selection, 2.5 μg/ml tetracycline was used and for *Escherichia coli*, 100 μg/ml ampicillin was used.

### Construction of a *Rex* Mutant

pORI280 was used to construct a marker-less *rex* mutant (MA1200) in *S. pneumoniae* D39 (Kloosterman et al., 2006). Left and right flanking regions of *rex* were generated using primer pairs Rex-KO-1/ Rex-KO-2 and Rex-KO-3/Rex-KO-4. The PCR fragment of the left flanking region contained *EcoRI* site at its 5′ end, whereas the PCR fragment of the right flanking region had *BamHI* at its 3′ end. These PCR fragments were joined together through overlap extension PCR and then cloned into the *EcoRI*/*BamHI* sites of pORI280 in *E. coli* EC1000, yielding pMA1200. The pOR1280 integrative vector carries an erythromycin resistance gene to select for chromosomal integrants and an expressed *lacZ* gene (colonies blue on X-Gal) to score the loss of the plasmid integrated into the chromosome. To achieve selection for erythromycin resistance, transformation of *S. pneumoniae* D39 was performed with pMA1200. pORI280 relies on RepA for replication. Therefore, the transformation of *S. pneumoniae* D39 results in single cross-over integration of the construct into the chromosome. Many LacZ-positive erythromycin-resistant integrants were plated on X-Gal medium (without antibiotic selection) as individual cultures for 30–50 generations (culturing two to four times until stationary phase). This led to the selection for clones that are now without the integration due to a second recombination event. Only few of the colonies were both white and erythromycin sensitive, which suggests that the plasmid had been excised from the chromosome. The required mutation was present in about eighty percent of these white erythromycin-sensitive colonies, which was then confirmed by colony PCR and DNA sequencing.

Transformation was performed by growing cells first at 37°C without shaking until an OD_600_ of ∼0.1. Afterwards, 100 ng/μl of CSP1, 0.2% BSA, and 1mM CaCl_2_ were added to 1ml of the grown culture in the 1.5 ml Eppendorf tube. After incubating cells at 37°C for 10–12 minutes, the DNA mixture was added to the incubated cells and the cells were grown for another 90–120 minutes at 37°C. The cell pellet was recovered by centrifugation for 1 minute at 7000 RPM. The cell pellet was dissolved in the 50–100 μl medium and plated out on blood agar plates.

### *lacZ*-Fusions Construction of Rex-Regulated Genes Promoters and Enzyme Assays

We used primer pairs mentioned in Supplementary Table S2 to construct chromosomal transcriptional *lacZ*-fusions to P*adhB2-lacZ*, P*gapN*-*lacZ*, P*gap-lacZ*, P*pncB-lacZ*, P*fba-lacZ*, and P*adhE-lacZ* in pPP2 (Halfmann et al., 2007), resulting in strains listed in Supplementary Table S1. The above-mentioned promoter fusions were also transformed into the D39 Δ*rex* strain, creating strains MA1201-MA1206, respectively. The following clones of P*gapN-lacZ* P*pncB-lacZ*, P*fba-lacZ*, P*gap-lacZ*, P*adhE-R1-lacZ*, and P*adhE-R2-lacZ* were constructed in pPP2 (Halfmann et al., 2007) with the primer pairs listed in Supplementary Table S2: P*fba-M*, P*pncB-M*, P*gap-M*, P*adhE-M-R1*, P*adhE-M-R2*, and P*gapN-M*, creating plasmids pMA1201-06, respectively. These *lacZ*-fusion constructs were transformed into the D39 wild-type, forming strains MA1207-12, respectively.

Beta-galactosidase assays were performed as mentioned previously using cells grown in CDM with or without added 0.5 mg/ml NADH and collected in the mid-log phase (Israelsen et al., 1995; Halfmann et al., 2007).

### DNA Microarray Analyses

Microarray comparison of *S. pneumoniae* (in replicates) was performed by growing *S. pneumoniae* D39 wild-type in CDM having 0 mg/ml NADH and 0.5 mg/ml NADH. CDM was prepared without niacin, because niacin is a precursor of NAD and NAD^+^. Similar procedure was performed for Δ*rex* where the global gene expression changes in Δ*rex* were measured by comparing the *S. pneumoniae* D39 Δ*rex* transcriptome against the *S. pneumoniae* D39 wild-type transcriptome both grown in replicates in complete CDM. Cells growing in the respective mid-log phase were collected for experiments. Data analysis and other procedures were adopted from previous studies (Shafeeq et al., 2011a,b; Afzal et al., 2015). A gene qualified to be differentially expressed if it satisfied a Bayesian *p*-value of <0.001 and a fold-change cut-off >1.5. Microarray data of this study submitted to Gene Expression Omnibus has been assigned the accession number GSE94573.

## Results

### NADH-Mediated Transcriptomic Response in *S. pneumoniae* D39

Niacin is a naturally existing vitamin B complex that forms important coenzymes NAD and NAD^+^, and also has a part in electron transfer in different metabolic activities (Wei et al., 2014). NAD is required in several enzymatic processes where it acts as an electron carrier either by being oxidized (NAD^+^) or reduced (NADH) (Belenky et al., 2007). Microarray comparison of *S. pneumoniae* was performed, where *S. pneumoniae* D39 wild-type was grown in CDM having 0 mg/ml NADH and 0.5 mg/ml NADH. Niacin was excluded from the CDM. Several genes/gene clusters exhibited differential expression under our experimental settings (**Table 1**). *spd-0113-124* were considerably downregulated when there was no NADH in medium. Several genes of this putative gene cluster (*spd-0113-124*) were also differentially expressed in our previous study, where we had explored the global gene expression changes induced by niacin (Afzal et al., 2017). Further investigation of the role of these genes in the presence of NADH and niacin might be very interesting.

**Table 1:** List of genes differentially expressed in *S. pneumoniae* D39 wild-type grown in CDM with 0 mg/ml to 0.5 mg/ml NADH.

D39 tag^a^	Function^b^	Ratio^c^
**Upregulated genes**		
*spd_0093*	Hypothetical protein	3.6
*spd_0094*	Hypothetical protein	2.6
*spd_0095*	Hypothetical protein	2.0
*spd_0474*	Hypothetical protein	1.8
*spd_0475*	CAAX amino terminal protease family protein	1.7
*spd_0526*	Fructose-1,6-bisphosphate aldolase, class II, Fba	1.9
*spd_1004*	Glyceraldehyde-3-phosphate dehydrogenase, NADP-dependent, GapN	1.8
*spd_1091*	Substrate-specific component predicted niacin ECF transporter, NiaX	2.1
*spd_1093*	Transcriptional regulator, biotin repressor family protein, NiaR	1.6
*spd_1251*	Nicotinate phosphoribosyltransferase, putative, PncB	1.7
*spd_1640*	Ribosyl nicotinamide transporter, PnuC-like, PnuC	3.4
*spd_1823*	Glyceraldehyde-3-phosphate dehydrogenase, type I, Gap	2.7
*spd_1824*	Hypothetical protein	2.2
*spd_1826*	Nicotinate-nucleotide pyrophosphorylase, NadC	4.1
*spd_1827*	Hypothetical protein	3.2
*spd_1834*	Alcohol dehydrogenase, iron-containing, AdhE	1.7
*spd_1864*	Hypothetical protein	1.8
*spd_1865*	Alcohol dehydrogenase, zinc-containing, AdhB2	1.5
*spd_1874*	LysM domain protein	2.4
**Downregulated genes**		
*spd_0113*	Hypothetical protein	–4.6
*spd_0114*	Hypothetical protein	–5.3
*spd_0115*	Hypothetical protein	–4.9
*spd_0116*	Hypothetical protein	–2.3
*spd_0117*	Hypothetical protein	–2.5
*spd_0118*	Hypothetical protein	–3.7
*spd_0119*	Hypothetical protein	–3.0
*spd_0120*	Hypothetical protein	–3.1
*spd_0121*	Hypothetical protein	–2.4
*spd_0122*	Hypothetical protein	–3.2
*spd_0123*	Hypothetical protein	–2.9
*spd_0124*	Hypothetical protein	–2.7
*spd_0936*	Tn5252, relaxase	–5.6

*^*a*^Gene numbers refer to D39 locus tags. ^*b*^D39 annotation (Lanie et al., 2007), ^*c*^Ratio denotes the fold increase/decrease in the expression of genes in CDM with 0 mg/ml to 0.5 mg/ml NADH.*

An increase in the expression of *spd-0093-95* could be observed in the absence of NADH. This putative gene cluster codes for membrane proteins of unknown functions. Another gene coding for a putative fructose-1,6-bisphosphate aldolase was upregulated in our tested conditions. The genes, putatively involved in the transport and metabolism of niacin, were significantly upregulated in the absence of NADH. These genes are *pnuC, niaX*, and *nadC*, and code for a ribosyl nicotinamide transporter, a predicted substrate-specific niacin ECF transporter, and a nicotinate-nucleotide pyrophosphorylase, respectively. NadC has been anticipated to convert quinolinate made from tryptophan, aspartate, alanine, and glutamate metabolism into nicotinate D-ribonucleotide (Kanehisa et al., 2014). Many other genes showed altered expression in the absence of NADH. These genes include *fba, pncB, gapN, gap, adhB2*, and *adhE*. The expression of these genes was also altered in the presence of niacin (Afzal et al., 2017). These genes are proposed to be involved in the NADH pathway. Therefore, we further investigated the regulatory mode of these genes in the presence of NADH.

### NADH-Mediated Expression of *gapN, fba, niaX, pnuC, pncB, gap, adhB2, adhE*, and *nadC*

**FIGURE 1:**
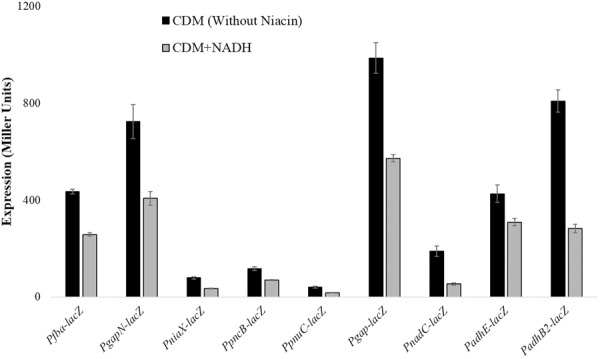
Expression levels (in Miller units) of P*gapN-lacZ*, P*fba-lacZ*, P*niaX-lacZ*, P*pnuC-lacZ*, P*pncB-lacZ*, P*gap-lacZ*, P*adhB2-lacZ*, P*nadC-lacZ*, and P*adhE-lacZ* in *S. pneumoniae* D39 wild-type grown in CDM with 0 mg/ml and 0.5 mg/ml NADH. CDM was prepared without niacin. Expression levels measurements were performed from three independent experiments and the values represented are means ± SD.

Beta-galactosidase activity for *pncB, gapN, fba, niaX, pnuC, gap, adhB2, adhE*, and *nadC* promoters *lacZ*-fusions made in *S. pneumoniae* D39 wild-type was measured using β-galactosidase assays. β-galactosidase assays results strengthened our microarray results and established that the expression of P*pncB*-*lacZ*, P*gapN*-*lacZ*, P*gap*-*lacZ*, P*fba-lacZ*, P*niaX*-*lacZ*, P*pnuC*-*lacZ*, P*nadC*-*lacZ*, P*adhE*-*lacZ*, and P*adhB2*-*lacZ* elevated notably without added NADH in the medium (**Figure 1**).

### Transcriptomic Analysis of D39 Δ*rex*

We further deleted the *rex* gene to examine its role in *S. pneumoniae* D39. Microarray study was performed, where *S. pneumoniae* D39 Δ*rex* transcriptome was compared to that of D39 wild-type grown in complete CDM. Niacin is present at the concentration 8 μM in complete CDM, which is the normal concentration of niacin used in preparing CDM. **Table 2** enlists the gene expression changes due to the absence of *rex* in *S. pneumoniae* D39. Expression of *fba, pncB, gapN, gap, adhB2*, and *adhE*, was significantly increased in D39 Δ*rex* signifying the role of Rex as a transcriptional repressor of *gapN, fba, pncB, adhE, adhB2*, and *gap* in *S. pneumoniae* D39.

**Table 2:** Genes differentially expressed in *S. pneumoniae* D39 Δ*rex* compared to the D39 wild-type grown in complete CDM.

D39 tag^a^	Function^b^	Ratio^c^
*spd_0526*	Fructose-1,6-bisphosphate aldolase, class II, Fba	2.9
*spd_0974*	Class I glutamine amidotransferase, putative, GuaA	2.4
*spd_0976*	Redox-sensitive transcriptional regulator Rex	–2.3
*spd_1004*	Glyceraldehyde-3-phosphate dehydrogenase, NADP-dependent, GapN	4.2
*spd_1250*	NAD^+^ synthetase, NadE	1.6
*spd_1251*	Nicotinate phosphoribosyltransferase, putative, PncB	2.0
*spd_1823*	Glyceraldehyde-3-phosphate dehydrogenase, type I, Gap	1.6
*spd_1834*	Alcohol dehydrogenase, iron-containing, AdhE	3.6
*spd_1865*	Alcohol dehydrogenase, zinc-containing, AdhB2	1.7

*^*a*^Gene numbers refer to D39 locus tags. ^*b*^D39 annotation (Lanie et al., 2007), ^*c*^Ratio signifies the fold increase/decrease in the expression of genes in D39 Δ*rex* compared to the D39 wild-type in complete CDM.*

### Rex Acts as a Transcriptional Repressor of *gapN, fba, gapN, pncB, adhE, adhB2*, and *gap*

**FIGURE 2:**
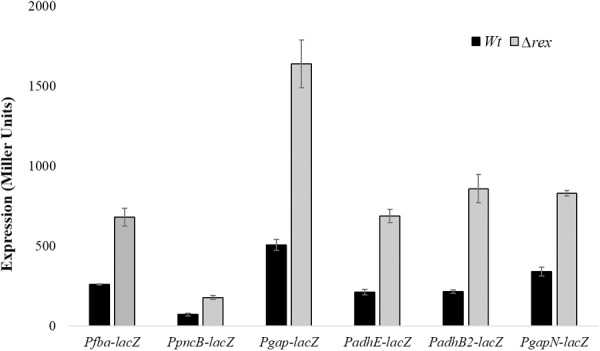
Expression levels (in Miller units) of P*fba-lacZ*, P*pncB-lacZ*, P*gap-lacZ*, P*adhE-lacZ*, P*adhB2-lacZ*, and P*gapN-lacZ* in *S. pneumoniae* D39 wild-type and D39 Δ*rex* grown in complete CDM. Expression levels measurements were performed from three independent experiments and the values represented are means ± SD.

To further investigate the role of Rex in the modulation of *gapN, fba, pncB, adhE, adhB2*, and *gap*, we constructed *lacZ* promoter fusions of *gapN, fba, pncB, adhE, adhB2*, and *gap* into *S. pneumoniae* D39 Δ*rex* and measured β-galactosidase activity in complete CDM (**Figure 2**). Our enzyme assays results indicated an upregulation in the expression of all these promoters *lacZ*-fusions in D39 Δ*rex* compared to the D39 wild-type, corroborating the role of Rex as a transcriptional repressor of *gapN, fba, pncB, adhE, adhB2*, and *gap*.

### Rex Recognition Site Prediction and Verification in P*gapN*, P*gap*, P*fba*, P*pncB*, P*adhB2*, and P*adhE*

**FIGURE 3:**
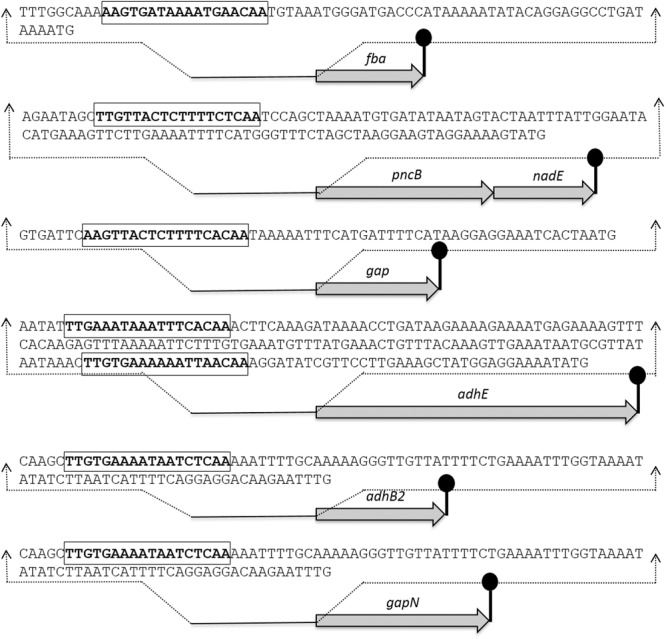
Detailed promoter sequences and organization of the Rex regulated genes in *S. pneumoniae* D39. Putative transcriptional terminators are denoted by the lollipop structures and the putative Rex operator sequences are in bold and rectangle.

The Rex sites are predicted in bacteria using a comparative genomics approach (Ravcheev et al., 2012). The prediction identifies two other putative Rex targets (*forT*/*spd*_*1075* and *hemH*/*spd*_*0895*) as well. A notable change in the expression of *forT* and *hemH* could not be established in our study neither in the presence of added NADH nor in the *rex* mutant. This might disprove the original prediction for these two genes. Moreover, Regprecise database has also speculated the Rex binding sites in *S. pneumoniae* (Novichkov et al., 2010). We analyzed the promoter regions of *gapN, adhB2, fba, gap, pncB*, and *adhE* using a software Genome-2D, and found an 18-bp palindromic-like sequence (5′- TTGTKAWAAWWTTCACAA -3′) similar to the Rex site projected by the Regprecise (Baerends et al., 2004; Novichkov et al., 2010). The Rex site found in the regulatory regions of *gapN, adhB2 fba, gap, pncB*, and *adhE* is shown in **Figure 3**.

**FIGURE 4:**
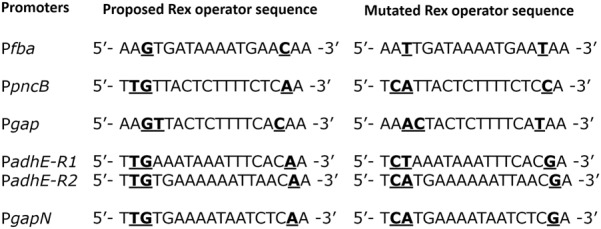
Aligned mutated and non-mutated Rex operator sequences in P*fba*, P*pncB*, P*gap*, P*adhE*, and P*gapN*.

**FIGURE 5:**
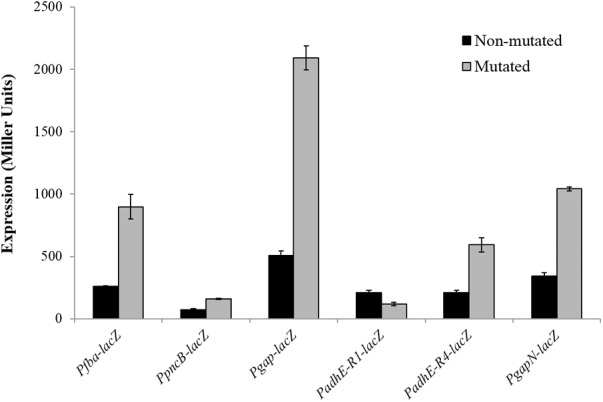
Expression levels (in Miller units) of P*fba-lacZ*, P*pncB-lacZ*, P*gap-lacZ*, P*adhE-lacZ*, and P*gapN-lacZ* with mutated and non-mutated Rex operator sites in *S. pneumoniae* D39 wild-type grown in complete CDM. Expression levels measurements were performed from three independent experiments and the values represented are means ± SD.

To verify the Rex operator site situated in the regulatory sequences of *gapN, adhB2, fba, gap, pncB*, and *adhE*, we made transcriptional *lacZ***-**fusions to P*fba*, P*pncB*, P*gap*, P*adhE*, and P*gapN*, in which consensus bases in the predicted Rex binding sites were mutated in P*fba*, P*pncB*, P*gap*, P*adhE*, and P*gapN* (**Figure 4**). We performed β-galactosidase assays in complete CDM. The expression of P*fba*, P*pncB*, P*gap*, and P*gapN* elevated considerably when Rex operator sites were mutated in the wild-type. This asserts that in *S. pneumoniae* D39, the predicted Rex sites in the regulatory areas of *fba, pncB, gap*, and *gapN* are intact and active (**Figure 5**). There are four possible operator sites for Rex in P*adhE*. Two of them were mutated individually in P*adhE* and β-galactosidase assays were performed. Derepression caused by Rex could only be seen when the Rex operator site R2 (5′- TTGTGAAAAAATTAACAA -3′) was mutated and we did not detect any significant change in the expression of P*adhE* due to mutation in Rex operator site R1 (5′- TTGAAATAAATTTCACAA -3′) (**Figure 4**). These data propose that operator site 2 (R2) is a functional operator site for Rex in P*adhE* (**Figure 5**). These data also suggest that the *in silico* predicted consensus sequences may not always be exactly correct and they must be confirmed by experiments.

## Discussion

Based on the available knowledge, NAD metabolism can be best termed as a chain of events, none of which are absolutely preserved (Gazzaniga et al., 2009). Moreover, we note that the modulation mechanisms regulating the expression of specific gene units and link NAD absorption to vibrant cellular and ecological exercises, are vaguely known. Advances in computational techniques, together with molecular genetic methods developed recently can be employed to monitor how NAD metabolic gene products and enzymes are modulated to react to environmental cues and to manage microbiological tasks (Gazzaniga et al., 2009). This study sheds light on the regulatory role of Rex in the transcriptional regulation of *adhB2, fba, pncB, gapN, adhE*, and *gap.*

Transcriptomic analysis of D39 wild-type with 0 mg/ml to 0.5 mg/ml NADH exhibited an increase in the expression of *niaX, pnuC, nadC, fba, pncB, gapN, gap, adhB2*, and *adhE*. Promoter *lacZ*-fusions and microarray studies with a *rex* mutant showed that Rex acts as a transcriptional repressor of several genes/operons (*gapN, fba, gap, pncB, adhB2*, and *adhE*) involved in niacin uptake and biosynthesis. Fba (a fructose-bisphosphate aldolase encoded by *fba*) has been deemed essential for glycolysis and gluconeogenesis and is a potential drug target in *Mycobacterium tuberculosis*. Fba has been the center of structural, enzymatic, and drug developmental studies (Pegan et al., 2009; Puckett et al., 2014). *gapN* encodes a glyceraldehyde-3-phosphate dehydrogenase and NAD-dependent glyceraldehyde-3-phosphate dehydrogenase, and *gap* encodes a glyceraldehyde-3-phosphate dehydrogenase. Glycolysis and gluconeogenesis employ GAPs as the key players by enhancing the reversible oxidative phosphorylation of D-glyceraldehyde 3-phosphate to the energy-rich intermediate glyceraldehyde 1,3-bisphosphate. Moreover, GAP is widely known to have an extensive range of biological functions (Zhou et al., 2008; Sirover, 2012). Extracellular GAPs are described to have a role in pathogenesis of many bacteria (Seifert et al., 2003; Oliveira et al., 2012). AdhE (an iron-containing alcohol dehydrogenase coded by *adhE* has been shown to increase pneumolysin (Ply) during the ethanol stress conditions, therefore, enhancing pneumococcal virulence (Luong et al., 2015). AdhE has also been shown to regulate virulence in *E. coli* (Beckham et al., 2014). *pncB* encodes a nicotinate phosphoribosyl-transferase and *adhB2* encodes a zinc-containing alcohol dehydrogenase. Involvement of the above-mentioned genes in virulence and their pivotal role for bacterial cell functions underscore the importance of studying their regulatory mode.

Our study demonstrates that in *S. pneumoniae* Rex is a transcriptional repressor of *gapN, adhB2, fba, pncB, adhE*, and *gap*. The Rex protein has been identified in numerous model microorganisms, including *S. aureus, B. subtilis, E. faecalis*, and *S. mutans*. In *B. subtilis*, Rex has been shown to repress expression of *cydABCD* (cytochrome bd oxidase), *yjlC*-*ndh* (NADH dehydrogenase), *ywcJ* (a formate-nitrate transporter), and *lctP*-*ldh* (NADH-linked fermentative lactate dehydrogenase) (Larsson et al., 2005; Gyan et al., 2006; Wang et al., 2008). Rex directly regulates at least 19 genes in *S. aureus*. It acts as a key transcriptional regulator of anaerobic metabolism leading to anaerobic NAD^+^ regeneration, which includes lactate, formate, and ethanol fermentation (*adh1, adhE, lctP, ldh1*, and *pflBA*) and nitrate respiration (*narG, nirC*, and *nirR*). Furthermore, in many organisms, Rex-mediated transcriptional regulation has been attributed to species-specific genetic targets (Ravcheev et al., 2012). Moreover, Rex regulates the transcription of genes involved in fermentation, oxidative stress response, and biofilm formation in cariogenic *S. mutans* (Bitoun et al., 2011, 2012; Baker et al., 2014). Based on RegPrecise database annotation^1^, it appears that the number of genes regulated by Rex can vary in different organisms. In addition to *gap, pncB*, and *adhE*, Rex may also be regulating *pgk* (phosphoglycerate kinase), *ldh* (lactate dehydrogenase), *fba* (Fructose-bisphosphate aldolase), *eno* (Enolase), and *frdC* (fumarate reductase) in *S. mutans* (Novichkov et al., 2010). Similarly, in addition to *gap, pncB*, and *adhB1*, Rex may be regulating *tpi* (triosephosphate isomerase), *noxE* (NADH oxidase), *ldh* (lactate dehydrogenase), *ahpC* (alkyl hydroperoxide reductase), *forT* (Formate/nitrite family of transporters), and *eno* (enolase) in *Streptococcus pyogenes* (Novichkov et al., 2010).

Interestingly, the binding sequence of Rex is highly conserved in Gram-positive bacteria. The reported consensus sequence in *S. coelicolor* (5′-TGTGAACNNNTTCACA-3′) (Brekasis and Paget, 2003), *B. subtilis* (5′-WWTGTGAANTNNTNNNCAAW-3′; W represents either A or T) (Wang et al., 2008), and *S. aureus* (5′-TTGTGAAWWWWTTCACAA-3′) (Pagels et al., 2010) are very similar. The binding site of Rex found in *S. pneumoniae* D39 (5′-TTGTKAWAAWWTTCACAA-3′) appears to be consistent with the above ones. Four putative Rex binding sites, 5′-TTGAAATAAATTTCACAA-3′, 5′-ATGAGAAAAGTTTCACAA-3′, 5′-TTATGAAACTGTTTACAA-3′ and 5′-TTGTGAAAAAATTAACAA-3′ were predicted to be preset in the *adhE* regulatory region. The first two sites and the last site are characterized by a high score (strong sites), whereas the third site is rather weak. The first and last sites were mutated in this study to check the functionality of the predicted sites and it was only the last site that appeared to be the functional operator site for Rex in the *adhE* regulatory region.

The expression of *niaX, pnuC, nadC, gapN, fba, pncB, adhE, adhB2*, and *gap* was also altered in our previous niacin microarray experiment, where we compared *S. pneumoniae* transcriptome with 0 mM to 10 mM niacin (Afzal et al., 2017). These genes are proposed to be involved in the NADH pathway, and niacin is the precursor of NADH. Proteins coded by two of the above-mentioned genes (NiaX and PnuC) are transporters (Johnson et al., 2015). We were unable to find a considerable difference in the expression of some other transporters, which might suggest that NiaX/PnuC might have a role in the transport of NADH. We are not sure whether NADH is potentially transported like niacin or whether it is first hydrolyzed to niacin extracellularly and then transported via NiaX/PnuC. One possible explanation might be that NADH is first converted into a form that is transportable by NiaX/PnuC. Although the data in literature show that NiaX exists as a preferred transporter for importing nicotinic acid and nicotinamide, whereas PnuC specifically imports nicotinamide ribosides (Johnson et al., 2015), NiaX and PnuC may be assisted by an extracellular protein having the ability to modify nicotinamide mononucleotide to an importable metabolite or pneumococcus might be possessing another import system (Johnson et al., 2015). The presence of another couple of putative niacin transporters (NiaY and NiaP) in the genomes of the Bacillus/Clostridium might support the theory of another possible niacin transporter in *S. pneumoniae* (Rodionov et al., 2008).

## Author Contributions

MA, SS, and OK substantially contributed to the conception or design of the work or the acquired, analyzed, or interpreted the data for the work; drafted the work or revised it critically for important intellectual content; gave final approval of the version to be published; and agreed to be accountable for all aspects of the work in ensuring that questions related to the accuracy or integrity of any part of the work are appropriately investigated and resolved.

## Conflict of Interest Statement

The authors declare that the research was conducted in the absence of any commercial or financial relationships that could be construed as a potential conflict of interest.

## Footnotes

1 http://regprecise.lbl.gov/RegPrecise/
