# Supplementary material for: NADH-Mediated Gene Expression in Streptococcus pneumoniae and Role of Rex as a Transcriptional Repressor of the Rex-Regulon
Source: Front Microbiol. 2018 Jun 19;9:1300. doi: 10.3389/fmicb.2018.01300 (PMC6018154; doi:10.3389/fmicb.2018.01300)
Supplement: Supplementary file 1 [file Table_1.PDF]

Table S1: List of strains and plasmids used in this study.

Strain/plasmid	Description	Source
S. pneumoniae		
D39	Serotype 2 strain. *cps 2*	Laboratory of P. Hermans.
MA1200	D39 Δ*rex* null mutant	This study
MA1301	D39 Δ*bgaA*:: P*niaX-lacZ*; Tet^R	(Afzal et al., 2017)
MA1302	D39 Δ*bgaA*:: P*pnuC-lacZ*; Tet^R	(Afzal et al., 2017)
MA1303	D39 Δ*bgaA*:: P*nadC-lacZ*; Tet^R	(Afzal et al., 2017)
MA1311	D39 Δ*bgaA*:: P*fba-lacZ*; Tet^R	(Afzal et al., 2017)
MA1313	D39 Δ*bgaA*:: P*gapN-lacZ*; Tet^R	(Afzal et al., 2017)
MA1314	D39 Δ*bgaA*:: P*pncB-lacZ*; Tet^R	(Afzal et al., 2017)
MA1315	D39 Δ*bgaA*:: P*gap-lacZ*; Tet^R	(Afzal et al., 2017)
MA1316	D39 Δ*bgaA*:: P*adhE-lacZ*; Tet^R	(Afzal et al., 2017)
MA1317	D39 Δ*bgaA*:: P*adhB2-lacZ*; Tet^R	(Afzal et al., 2017)
MA1201	MA1200 Δ*bgaA*:: P*fba-lacZ*; Tet^R	This study
MA1202	MA1200 Δ*bgaA*:: P*gapN-lacZ*; Tet^R	This study
MA1203	MA1200 Δ*bgaA*:: P*pncB-lacZ*; Tet^R	This study
MA1204	MA1200 Δ*bgaA*:: P*gap-lacZ*; Tet^R	This study
MA1205	MA1200 Δ*bgaA*:: P*adhE-lacZ*; Tet^R	This study
MA1206	MA1200 Δ*bgaA*:: P*adhB2-lacZ*; Tet^R	This study
MA1207	D39 Δ*bgaA*:: P*fba-M-lacZ*; Tet^R	This study
MA1208	D39 Δ*bgaA*:: P*pncB-M-lacZ*; Tet^R	This study
MA1209	D39 Δ*bgaA*:: P*gap-M-lacZ*; Tet^R	This study
MA1210	D39 Δ*bgaA*:: P*adhE1-M-lacZ*; Tet^R	This study
MA1211	D39 Δ*bgaA*:: P*adhE2-M-lacZ*; Tet^R	This study
MA1212	D39 Δ*bgaA*:: P*gapN-M-lacZ*; Tet^R	This study
E. coli		
EC1000	Km^R; MC1000 derivative carrying a single copy of the pWV1 *repA* gene in *glgB*	Laboratory collection
**Plasmids**		
pPP2	Amp^R Tet^R; promoter-less *lacZ*. For replacement of *bgaA* with promoter *lacZ* fusion. Derivative of pPP1	(Halfmann et al., 2007)
pORI280	Erm^R; *ori*$^+$ *repA*$^-$*;* deletion derivative of pWV01; constitutive *lacZ* expression from P32 promoter	(Leenhouts et al., 1998)
pMA1301	pPP2 P*niaX*	(Afzal et al., 2017)
pMA1302	pPP2 P*pnuC*	(Afzal et al., 2017)
pMA1303	pPP2 P*nadC*	(Afzal et al., 2017)
pMA1308	pPP2 P*fba*	(Afzal et al., 2017)
pMA1309	pPP2 P*rex*	(Afzal et al., 2017)
pMA1310	pPP2 P*gapN*	(Afzal et al., 2017)
pMA1311	pPP2 P*pncB*	(Afzal et al., 2017)
pMA1312	pPP2 P*gap*	(Afzal et al., 2017)

pMA1313	pPP2 P*adhE*	(Afzal et al., 2017)
pMA1314	pPP2 P*adhB2*	(Afzal et al., 2017)
pMA1200	pORI280 carrying *rex* deletion	This study
pMA1201	pPP2 P*fba-M*	This study
pMA1202	pPP2 P*pncB-M*	This study
pMA1203	pPP2 P*gap-M*	This study
pMA1204	pPP2 P*adhER1-M*	This study
pMA1205	pPP2 P*adhER2-M*	This study
pMA1206	pPP2 P*gapN-M*	This study
