# Supplementary material for: NADH-Mediated Gene Expression in Streptococcus pneumoniae and Role of Rex as a Transcriptional Repressor of the Rex-Regulon
Source: Front Microbiol. 2018 Jun 19;9:1300. doi: 10.3389/fmicb.2018.01300 (PMC6018154; doi:10.3389/fmicb.2018.01300)
Supplement: Supplementary file 2 [file Table_2.PDF]

Table S2: List of primers used in this study.

Name	Nucleotide Sequence (5'→3')	Restrictio
Rex-KO-1	CATGGAATTCCCTCATGGATAGCTTGGTAG	*EcoRI*
Rex-KO-2	GCTGTAGCTTTTGGAATAGC	-
Rex-KO-3	CCCTCCTCTACTTCATGCGGCTATTCCAAAAGCTACAGC	-
Rex-KO-4	CATGGGATCCCCAATGGTCTTCGATATCTTGG	*BamHI*
fba-F	CATGGAATTCCGTCCAAGACTAGGGAGAG	*EcoRI*
fba-R	CATGGGATCCGCATAACCGTTGTCACGGG	*BamHI*
gapN-F	CATGGAATTCGGTTTGGCTGTCCCCAACC	*EcoRI*
gapN-R	CATGGGATCCGTCATGGCTGGAACTGTACC	*BamHI*
pncB-F	CATGGAATTCGCTATGGCGAATGGGCTC	*EcoRI*
pncB-R	CATGGGATCCCTGGTACAAGTCCGTGTGC	*BamHI*
gap-F	CATGGAATTCCGTTACGCTATGAATAATAAGGG	*EcoRI*
gap-R	CATGGGATCCCGACCGATACGTCCGAAACC	*BamHI*
adhE-F	CATGGAATTCGCGCTTACCTGTAAATCCC	*EcoRI*
adhE-R	CATGGGATCCGAACCAACTCATCTACGTGC	*BamHI*
adhB2-F	CATGGAATTCGCAACCTACCTAGATGGCG	*EcoRI*
adhB2-R	CATGGGATCCGCACAATAGCGTCTGTTGGC	*BamHI*
fba-M-R	CATGGGATCCGCCATTTTATCAGGCCTCCTGTATATTTTTATGGGTCAT CCCATTTACATTATTCATTTTATCAATTT	*BamHI*
pncB-M-F	CATGGAATTCGAGAATAGCTCATTACTCTTTTCTCCATCC	*EcoRI*
gap-M-R	CATGGGATCCCCATTAGTGATTTCCTCCTTATGAAAATCATGAAATTTT TATTATGAAAAGAGTAGTTTGAATC	*BamHI*
adhE-M-R1-F	CATGGAATTCAAAAATATTCTAAATAAATTTCACGA	*EcoRI*
adhE-M-R2-R	CATGGGATCCCCATATTTTCCTCCATAGCTTTCAAGGAACGATATCCTT CGTTAATTTTTTCATGA	*BamHI*
gapN-M-F	CATGGAATTCTCAAGCTCATGAAAATAATCTCGAAAATT	*EcoRI*
